# Editorial: Gateways to the brain: vascular-glial-immune network in health and disease

**DOI:** 10.3389/fncel.2024.1461604

**Published:** 2024-09-26

**Authors:** Diana Matias, Patrícia P. Garcez, Helena Florindo, Luis Graça, Luiz Gustavo Dubois

**Affiliations:** ^1^Instituto de Medicina Molecular João Lobo Antunes, Faculdade de Medicina, Universidade de Lisboa, Lisbon, Portugal; ^2^Institute of Psychiatry, Psychology & Neuroscience, King's College London, London, United Kingdom; ^3^Faculty of Pharmacy, University of Lisbon, Lisbon, Portugal; ^4^Institute of Biomedical Sciences, Federal University of Rio de Janeiro, Rio de Janeiro, Brazil

**Keywords:** blood brain barrier (BBB), glymphatic system, immune cells, glial cells, neurons, oligodendrocytes, neuro disorders, brain tumors

The brain, a vital organ, is safeguarded by biological barriers such as the blood-brain barrier (BBB), which regulates the exchange of compounds between the blood and the brain, protecting it from pathogens and drugs. The gut-brain axis and gut microbiota are essential for maintaining the BBB, which can influence brain health. Understanding these networks is crucial for developing new treatments for brain disorders. This Research Topic explores the brain-immunology axis and the gut-brain axis and their impact on neurodegenerative disorders such as schizophrenia (SCZ), Alzheimer's disease (AD), Parkinson's disease (PD), and multiple sclerosis (MS) and brain tumors such as meningiomas. Among all the submissions, we have selected manuscripts with scientific merit, including original research and reviews.

Ben-Azu et al. explore how gut-resident bacteria can potentially influence brain function and contribute to age-related neurodegenerative disorders such as SCZ. The study highlights the significant role of environmental factors in altering the microbiota, including birth mode, diet, stress, pollution, and infections. These alterations affect neuronal activity through the vagus nerve and the enteric nervous system. The review reveals how microbiota-derived molecules can modulate microglia, the brain's immune cells, leading to brain remodeling, and aberrant plasticity from fetal development to early psychosis. Microglia are active participants in neurogenesis, synapse phagocytosis, and neurological dysfunction. The study emphasizes the gut-brain-microglia axis in SCZ pathology, showing the impact of environmental factors on the brain, accelerated aging, and the disruptive effects of gut dysbiosis on microglial function. It also examines the therapeutic and adverse effects of antipsychotics on the gut microbiome, the potential of fecal microbiota transplants, and the emerging field of psychobiotics. The authors suggest that psychobiotics could offer a promising new avenue for SCZ treatment by enhancing antipsychotic benefits, reducing adverse effects, and mitigating aging via the gut-brain-microglia axis. This research could significantly improve our understanding of SCZ pathogenesis related to chronobiology and the gut microbiome, potentially revolutionizing SCZ treatment approaches.

Aran et al. emphasize the use of liquid biopsy as a promising non-invasive technique for the diagnosis and monitoring of central nervous system (CNS) tumors, including meningiomas. They examined cfDNA from blood and fresh tumor samples using the ddPCR technique to identify mutations with low allelic frequencies. They studied plasma cytokines, highlighting their potential benefit for meningioma patients, particularly considering the various meningioma subtypes. Additionally, they explored miRNAs, which are detectable in body fluids such as blood, making them valuable cancer biomarkers for diagnosis and prognosis. They focused on miR-21, which is usually expressed in advanced meningioma tissue but had not been previously explored in blood, finding it in only some patients. To fully understand its clinical importance in meningiomas, more extensive research with large cohort studies is not just recommended but urgent.

Neto et al. explore the impact of obesity in AD, PD, and MS through multifaceted mechanisms that highlight the critical interplay between metabolic health and brain function. In AD, obesity worsens pathology by increasing amyloid precursor protein (APP) and beta-amyloid (Aß) levels, leading to synaptic loss, and cognitive decline. Despite elevated leptin levels in obesity, which could potentially reduce Aß accumulation, insulin resistance associated with obesity impairs insulin signaling pathways and reduces insulin receptor expression, thereby promoting Aß buildup. In PD, obesity accelerates α-synuclein aggregation, oxidative stress, and inflammation, resulting in the destruction of dopaminergic neurons crucial for motor function. Dysregulation of dopamine production enzymes like tyrosine hydroxylase (TH) further impairs dopaminergic function, exacerbating motor symptoms. Additionally, the downregulation of Peroxisome Proliferator-Activated Receptors (PPARs) in PD-related brain regions exacerbates neuroinflammation and oxidative stress, underscoring the intricate relationship between obesity and neurodegenerative processes. In MS, obesity worsens disease severity, correlating with higher relapse rates and accelerated disability progression. Early-life obesity increases MS risk later in life, highlighting the enduring impact of metabolic health on neurological outcomes. Metabolically, obesity alters the metabolism of disease-modifying therapies (DMTs), potentially compromising treatment efficacy. Obesity-induced oxidative stress heightens the vulnerability of oligodendrocytes, which are crucial for myelin maintenance, leading to increased myelin damage in MS. Obesity disrupts the BBB, allowing greater infiltration of pro-inflammatory immune cells such as Th1 and Th17 cells into the CNS, intensifying neuroinflammation. This inflammatory milieu, characterized by reduced regulatory T cells (Tregs) and elevated leptin levels in CNS lesions, exacerbates MS progression and complicates treatment strategies. Understanding these complex interactions between obesity, metabolic health, and neurodegenerative diseases is crucial for developing targeted interventions. Addressing obesity through lifestyle changes, diet, and medications not only improves overall health but also holds promise in mitigating its impact on neurological conditions. Further research is essential to uncover novel therapeutic approaches for managing neurodegenerative diseases in the context of metabolic dysfunction.

Sabaté San José and Petersen investigate the impact of melanocyte inducing transcription factor (MITF) loss on mast cells (MCs) in the meninges and their significance. This study demonstrates that MITF-null mice lack meningeal MCs, making them valuable for studying the role of MCs in CNS immune responses. However, research involving MITF-mutant mice must also consider the absence of meningeal melanocytes, considering the lack of both cell types in any hypotheses about MITF function in the CNS. The findings on MITF-heterozygous mice, which exhibit a reduction in meningeal MCs by about 25%, such as the 50% reduction observed in cardiac MCs, highlight the necessity for further research. This reduction suggests a tissue-dependent MITF dosage effect or maintenance defect in heterozygotes, consistent with known MITF haploinsufficiency. Given that increased inflammation is central to CNS pathologies and aging affects MCs, the use of MITF-heterozygous mice in studying meningeal MCs during aging could be transformative. However, further research is urgently needed to explore the impact of MITF heterozygosity on meningeal MC function during aging.

In summary, these studies highlight the crucial role of environmental factors and metabolic health in influencing neurological outcomes across diverse disorders, ranging from neurodegenerative diseases to cancer and immune responses. Moreover, these findings reinforce the profound interconnection between the gut-brain axis and BBB integrity, underscoring their significant influence on neurological health. Thus, gaining insight into these complex relationships deepens our understanding of disease mechanisms and paves the way for innovative therapies for and preventive strategies against challenging neurological conditions.

